# Mechanisms of Neural Stem Cell Mediated Neurogenesis and Therapeutic Prospects Following Ischemic Stroke: A Review

**DOI:** 10.1155/sci/6934557

**Published:** 2026-10-26

**Authors:** Shuhong Wang, Ji Zhang, Peng Wang, Peng Ye, Jin Wu, Lili Shi, Xuheng Jiang, Yuanlan Lu, Hongfei Ge, Anyong Yu

**Affiliations:** ^1^ Department of Emergency Affiliated Hospital of Zunyi Medical University Zunyi Guizhou China zmchospital.com.cn; ^2^ Department of Neurosurgery Southwest Hospital Third Military Medical University (Army Medical University) Chongqing China tmmu.edu.cn

**Keywords:** ischemic stroke, microenvironmental disturbance, neural stem cell, neurogenesis

## Abstract

Ischemic stroke is a refractory cerebrovascular disorder characterized by high incidence, disability, and mortality rates. Its core pathological features include extensive neuronal death within the ischemic zone and irreversible damage to neural circuits. Given the limited regenerative capacity of the central nervous system, regulating endogenous neurogenesis and reconstructing functional circuits are important strategies for treating neurological deficits. This narrative review summarizes the dynamic processes of activation, proliferation, migration, and differentiation of endogenous neural stem cells (NSCs) following ischemic stroke, and explores the therapeutic potential of exogenous NSC transplantation and its current applications. Distinct from existing reviews, this work systematically integrates neuroimmune crosstalk, metabolic reprogramming, and meningeal‑choroid‑plexus‑mediated niche regulation, further proposing a staged multimodal precision intervention framework. We also highlight critical confounding factors, including aging‑driven regenerative decline, and explicitly separate preclinical animal findings from limited human clinical evidence. Meanwhile, this review elucidates the core mechanisms through which neurogenesis mediates tissue repair and functional recovery in ischemic brain tissue. Furthermore, this review refines future research directions, such as spatiotemporally specific precision interventions and multimodal combination therapies, providing a systematic theoretical basis and research framework for developing novel NSC‐based therapeutic strategies for ischemic stroke.

## 1. Introduction

Ischemic stroke is a major cause of death and long‐term disability worldwide [1, 2]. Its core pathological feature is irreversible neuronal damage resulting from an interrupted cerebral blood flow. Neurons within the ischemic zone undergo apoptosis and necrosis owing to hypoxia and impaired energy metabolism. Given the limited regenerative capacity of the central nervous system, neuronal loss leads to permanent neurological deficits [3, 4]. Reperfusion therapies such as intravenous tissue plasminogen activator (tPA) thrombolysis and mechanical thrombectomy are primary interventions for acute ischemic stroke. Although these interventions rapidly restore blood supply to the ischemic area, their capacity to repair neural damage remains limited [4, 5]. Furthermore, constraints such as a narrow therapeutic time window and an increased risk of intracranial hemorrhage hinder fundamental improvement in neurological outcomes poststroke. Consequently, achieving neural damage repair and promoting neurofunctional reconstruction have emerged as core research focuses in this field. Endogenous neural stem cells (NSCs) located in the subventricular zone (SVZ) of the lateral ventricles and the subgranular zone (SGZ) of the dentate gyrus in the adult mammalian brain possess self‐renewal and multipotent differentiation capacities and can give rise to new neurons. This phenomenon establishes the cellular basis for endogenous neural repair after stroke [3, 6]. Cerebral ischemic injury activates endogenous neurogenesis through promoting NSC migration toward the lesion site and differentiation into mature neurons for functional recovery [7]. However, the reparative capacity of endogenous NSC is limited, failing to achieve complete repair [3]. Consequently, exogenous NSC transplantation aimed at replenishing functional neurons has emerged as a promising strategy for brain repair poststroke. Stem cell therapy, which is primarily categorized into exogenous NSC transplantation and endogenous NSC activation, holds significant promise for clinical translation. Recent studies have identified several pathways, including Wnt/β‐catenin and PI3K/Akt pathways, as core regulators of neurogenesis [7, 8] and revealed the intricate regulatory networks of NSC niches. These networks involve immune cell polarization, metabolic reprogramming, and regulatory roles of the meninges and choroid plexus [9, 10]. Concurrently, interventions involving mesenchymal stem cells and their secretome alongside electroacupuncture have shown potential in promoting neurogenesis [11, 12], providing a crucial theoretical foundation and potential targets for developing neurogenesis‐based strategies after stroke.

Compared with exogenous stem cell transplantation, endogenous neurogenesis avoids the risks of immune rejection and tumorigenesis, thus exhibiting superior clinical translational safety. Although there has been increasing progress in research into the mechanisms of poststroke neurogenesis and related interventions, the low efficiency of endogenous neural repair and the limitations of existing regulatory strategies continue to hinder the clinical application of stem cell therapies in ischemic stroke. The complex interactions between signaling pathways, microenvironmental regulatory mechanisms, and external intervention methods also present unresolved challenges to optimizing neurogenesis efficiency and achieving stable neurological recovery. Against this backdrop, further in‐depth investigation into the key regulatory mechanisms of NSC‐mediated neurogenesis, alongside the exploration of highly effective and safe intervention methods, is crucial for overcoming the current bottlenecks in poststroke repair therapies and improving the prognosis of patients with ischemic stroke. Herein, we first dissect the multistage dynamic process of endogenous neurogenesis, then elaborate core signaling pathways and niche‐microenvironment regulatory networks, and summarize three categories of targeted therapeutic strategies. We further analyze major translational bottlenecks and propose staged multimodal precision intervention schemes for future research.

Recent studies have provided comprehensive reviews of the clinical translation of stem cells, immune‐stem cell interactions, and the therapeutic characteristics of neurogenesis following stroke. Representative prior reviews include Stem Cell Therapy for Stroke: Mechanisms, Clinical Translation, and Future Perspectives [13], How Neural Stem Cell Therapy Promotes Brain Repair After Stroke [14], Stem Cell‐Mediated Recovery in Stroke: Partnering with the Immune System [15], and Cerebral Stroke‐Induced Neurogenesis: Insights and Therapeutic Implications [16]. However, existing reviews have not systematically elucidated an integrated microenvironmental regulatory network encompassing neuroimmune interactions, metabolic reprogramming, and the regulation of the microenvironment by the meningeal‐choroid plexus. Furthermore, few studies have established a phased, precision intervention framework to bridge the gap between preclinical mechanistic discoveries and translational bottlenecks. Consequently, this review focuses on neurogenesis mediated by NSCs following ischemic stroke, integrating intracellular signaling cascades, multicomponent microenvironmental regulation, and core therapeutic strategies. This article proposes a phased, multimodal precision intervention workflow, focusing on key translational barriers, including the causal gap between neonatal neuronal integration and functional recovery, age‐related declines in regenerative capacity, and safety concerns regarding cell transplantation. This study provides a systematic and unique theoretical framework that complements current relevant reviews.

## 2. Dynamic Regulation of Endogenous Neurogenesis After Ischemic Stroke

Endogenous neurogenesis after ischemic stroke is a multistep, finely regulated process comprising three core phases: activation and proliferation of NSC, directed migration of newly generated NSC, and neuronal differentiation with functional integration. These phases interconnect and regulate each other, collectively determining the eventual efficiency of neurogenesis and the outcomes of functional recovery.

### 2.1. Activation and Proliferation of Quiescent NSC

Cerebral ischemic injury effectively activates quiescent NSC within neurogenic regions such as the SVZ and SGZ of the adult brain. This process induces the exit of these cells from the G0 phase into the cell cycle, initiating their proliferation. This process is accompanied by the upregulation of stem cell markers such as Nestin and Sox2 [17]. Growth factors such as fibroblast growth factor 2 (FGF‐2) and insulin‐like growth factor 1 (IGF‐1) promote the activation and proliferation of NSC. This proliferative response exhibits spatiotemporal specificity, peaking at 1 week after the injury. Cell proliferation is influenced by factors such as age and injury severity, with elderly individuals retaining neurogenic potential [17]. Following a stroke, proliferating NSCs/progenitor cells in the SVZ and SGZ exhibit elevated expression of Ki‐67 (an endogenous proliferation marker). Cell proliferation can also be quantified using BrdU labeling; BrdU is an exogenous thymidine analog used in animal experiments to track cells undergoing DNA synthesis [18]. It is worth noting that dysregulation of the fate determination of activated NSCs may lead to an unfavorable prognosis following injury. It should be emphasized that NSCs that fail to differentiate normally typically undergo apoptosis in vivo rather than directly forming glial scar tissue. Following ischemic stroke, reactive astrocytes (rather than the NSCs themselves) are the primary cellular source of glial scar formation. Uncontrolled proliferation of NSCs may indirectly exacerbate the pathological microenvironment through the release of pro‐inflammatory signals, thereby further promoting astrocyte proliferation and glial scar deposition, ultimately hindering the reconstruction of neural circuits [19, 20]. The Wnt/β‐catenin and Notch pathways constitute the core regulatory network governing NSC proliferation. The activation of the Wnt/β‐catenin pathway significantly promotes NSC proliferation, whereas the latter is involved in regulating proliferation [21–23]. In addition, exogenous mannitol exposure can suppress NSC proliferation, and Mysm1 gene deficiency also disturbs NSC proliferative homeostasis via separate molecular pathways [24, 25]. This intricate regulatory network offers potential targets for therapeutic intervention.

It should be noted that the majority of mechanistic observations regarding the proliferation of NSCs have been derived from rodent experimental models. There are significant interspecies differences between the neurogenic microenvironments of rodents and humans, and in vivo evidence from humans remains very limited.

### 2.2. Directed Migration and Target Site Accumulation of NSC

Neural progenitor cells/neuroblasts derived from NSC proliferation undergo directed migration toward the ischemic injury site. Under physiological conditions, neural progenitor cells from the SVZ migrate along the rostral migrating stream (RMS) toward the olfactory bulb. However, NSCs could migrate from the germinal zone to the infarct region after ischemic stroke [26]. The SDF‐1/CXCR4 axis is the core pathway directing NSC migration, and the physical scaffold formed by the extracellular matrix and reactive astrocytes provides structural support for migration [26, 27].

The pathological microenvironment exerts a dual effect on neural progenitor cell migration following stroke. Some chemotactic stimuli such as SDF‐1 and BDNF form concentration gradients that attract cells toward the injury site [27], whereas excessive inflammatory responses and glial scarring form physical and chemical barriers. In addition, microglia/macrophages within the ischemic zone can directly inhibit NSC migration [9]. The hyperpolarization‐activated cyclic nucleotide–gated channel 2 (HCN2) subunit promotes NSC migration by sensing hypoxic microenvironments [28]. The administration of Rac1 activators promotes NSC migration after stroke [29].

### 2.3. Neuronal Differentiation and Functional Integration of NSC

NPC migrating to the ischemic injury site holds the capacity for differentiation into mature neurons under microenvironmental signaling regulation. This process is governed by multiple signals, including neurotrophic factors and cytokines, with NSC differentiation into functional neurons, such as GABAergic and glutamatergic types [17, 30, 31]. The Wnt/β‐catenin pathway plays an essential role in cell differentiation, which drives the transformation of NSC into neurons [21, 22]. The long noncoding RNA Pnky also regulates cell differentiation by modulating this pathway [30]. The differentiation of NPC into neurons expressing mature markers is necessary but insufficient for achieving functional neural repair. The functional integration of these newly generated neurons is essential. This process requires establishing synaptic connections with host neurons and integrating new neurons into damaged neural circuits. This process involves dendritic spine formation and electrophysiological maturation. Its success directly determines the behavioral repair outcomes of neurogenesis [32]. Ginsenosides can enhance synaptic plasticity by activating the Akt/mTOR/p70S6K pathway [33], and the secretome derived from human NSC improves the inflammatory microenvironment by promoting microglia polarization [34]. These mechanisms promote the functional integration of newly generated neurons. However, the efficiency of achieving this integration remains a core challenge for neurogenic therapies (Figure 1).

**Figure 1:**
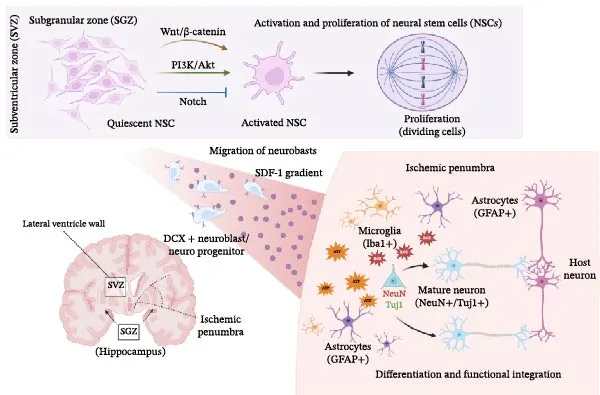
Schematic illustration of three consecutive and dynamically regulated phases of endogenous neurogenesis following ischemic stroke. Within the neurogenic microenvironment (SVZ and SGZ), quiescent neural stem cells (NSCs) are triggered, activated, and undergo proliferation: the Wnt/β‐catenin signaling cascade promotes proliferation, whilst the Notch signaling pathway exerts an inhibitory regulatory effect. Newly generated DCX‐positive neuroblasts migrate toward the ischemic penumbra along an SDF‐1 chemotactic gradient, but their migratory movement is inhibited by inflammatory microglia releasing ATP and ROS. Upon reaching the lesion, neural precursor cells differentiate into NeuN+/Tuj1+ mature neurons and form synaptic connections with resident neurons, thereby completing the integration of functional neural circuits. GFAP+ astrocytes and Iba1+ microglia jointly shape the microenvironment of the penumbra and limit the overall efficiency of endogenous neural repair.

## 3. Core Molecular Signaling Pathways Regulating Neurogenesis

Neurogenesis after ischemic stroke is regulated by a complex molecular signaling network. Wnt/β‐catenin and PI3K/Akt are two core regulatory pathways, and key signaling molecules such as Rac1, mTOR, and EphB4 play various roles in regulating neurogenesis. Extensive crosstalk exists between these pathways and molecules, forming a sophisticated regulatory network that guides the orderly progression of neurogenesis [21, 22, 35, 36].

### 3.1. Wnt/β‐Catenin Pathway: A Core Regulatory Axis of Neurogenesis

The Wnt/β‐catenin pathway is a classical regulator of NSC self‐renewal, proliferation, and differentiation, playing a central role in neurogenesis after stroke. Once a stroke occurs, this pathway undergoes rapid activation. β‐catenin translocates to the nucleus and initiates the transcription of downstream target genes such as cyclin D1 and neuroD1, thereby driving NSC proliferation and neuronal differentiation [7]. The activation of the Wnt/β‐catenin pathway regulates neural stem/progenitor cell regeneration in a dose‐dependent and cell‐state‐specific manner [37, 38]. Exogenous substances such as phenethyl alcohol glycosides, ambroxol, and kaempferol can activate the Wnt/β‐catenin pathway through various mechanisms, exerting neuroprotective effects by promoting neurogenesis and inhibiting apoptosis [39–41]. The Wnt/β‐catenin pathway extensively cross‐links with other signaling networks. For instance, Sbno1 converges with this pathway through the PI3K–Akt–GSK3β axis to regulate NSC proliferation [35]. Dkk3 acts as an atypical inhibitor of the Wnt/β‐catenin pathway, suppressing neurogenesis, whereas curcumin can reactivate this pathway by inhibiting Dkk3 [42]. Given the multifaceted benefits of the Wnt/β‐catenin pathway in promoting neurogenesis, angiogenesis, and anti‐inflammation, targeted modulation of this pathway is a promising therapeutic strategy for stroke, particularly during the recovery phase [43].

### 3.2. PI3K/Akt Pathway: Regulation of NSC Survival and Differentiation

The PI3K/Akt pathway plays a key role in regulating cell survival, proliferation, and metabolism. Its activation after stroke is necessary for inhibiting NSC apoptosis and promoting neuronal differentiation. It functions by phosphorylating and inhibiting downstream targets such as GSK3β, thereby integrating endogenous and exogenous signals to finely modulate neurogenesis. Aglycone IV and high‐frequency repetitive transcranial magnetic stimulation can promote neurogenesis by activating the PI3K/Akt pathway [35, 44]. GSK3β serves as an important intersection between the PI3K/Akt and Wnt/β‐catenin pathways. Its inactivation through phosphorylation is a common downstream event in both pathways, achieving a synergistic regulation of NSC survival and differentiation. The downstream targets of Akt, including mTOR and FOXO, respond to diverse cellular signals to participate in the multilevel regulation of neurogenesis. Heme oxygenase‐1 (HO‐1) overexpression inhibits the Wnt/β‐catenin pathway by suppressing the AKT/GSK3β pathway, decreasing the capacity of NSC proliferation [36]. Overall, the PI3K/Akt pathway activation not only provides survival and metabolic support for neurogenesis but also functions as a signaling hub involved in crosstalk with other pathways, thereby ensuring the progression of neural repair.

### 3.3. Key Signaling Nodes: Multidimensional Coordinated Regulation of Neurogenesis

In addition to the Wnt/β‐catenin and PI3K/Akt pathways, mediators such as Rac1, mTOR, and EphB4 play indispensable roles in neurogenesis poststroke. These molecules interact with the aforementioned core pathways in an interwoven, synergistic manner, promoting neural repair by regulating cell migration, metabolism, and axon guidance. Rac1 is a key regulator of cytoskeletal rearrangement and cell migration. Maintaining its activity is crucial for recruiting NSC to the injury site. In addition, Rac1 participates in vascular regeneration within the ischemic zone and synaptic remodeling, making it a potential target for promoting migration and synaptic restructuring [45]. The mTOR kinase integrates nutrient, energy, and growth factor signaling, globally regulating protein synthesis and autophagy. Its moderate activation enhances neurogenesis, angiogenesis, and synaptic plasticity, providing a biosynthetic foundation for NSC proliferation and differentiation and mature neuron development [44]. EphB4 and its ligand ephrinB2 are upregulated after stroke, promoting NSC proliferation and differentiation while suppressing neuroinflammation to exert dual restorative and anti‐inflammatory effects. Their signaling pathways are involved in crosstalk with the Wnt pathway during axon guidance, contributing to brain repair poststroke [46] (Figure 2).

**Figure 2:**
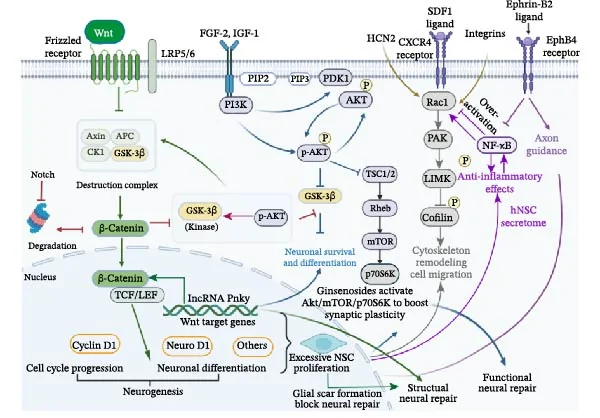
Schematic diagram of the multisignal network regulating endogenous neurogenesis following ischemic stroke. The core signaling cascades include Wnt/β‐catenin, PI3K/Akt/mTOR, Rac1‐PAK, and EphB4/NF‐κB, which synergistically regulate the proliferation, directed migration, and neuronal differentiation of neural stem cells (NSCs) under resting conditions, thereby mediating the structural and functional repair of the nervous system. Growth factors (FGF‐2, IGF‐1), the chemokine SDF‐1, the ion channel HCN2, the long noncoding RNA Pnky, the hNSC secretome, and ginsenosides all act as positive regulators of neurogenesis. In contrast, the Notch signaling pathway, glial scarring, hyperactivated NF‐κB, and microglia exert inhibitory effects on NSC‐mediated neural repair.

## 4. Multifactorial Regulatory Networks of NSC Niches

The biological behavior of NSC is regulated by both intracellular signaling pathways and extracellular NSC niches. The intracerebral microenvironment undergoes profound remodeling after stroke. Neuroimmune interactions, cellular metabolic reprogramming, and specific regulation by the meninges and choroid plexus collectively form a microenvironmental regulatory network governing neurogenesis, directly influencing the efficiency and direction of neurogenesis.

### 4.1. Neuroimmune Interactions: Bidirectional Regulatory Effects of Microglia/Macrophages

Microglia and infiltrating peripheral macrophages rapidly activated in the ischemic zone after stroke are the primary immune components. The dynamic of M1/M2 polarization exerts dual effects on neurogenesis. The activation of M1 inhibits ischemia‐induced neural stem/progenitor cell proliferation and differentiation through direct contact with or secretion of pro‐inflammatory factors such as IL‐1β and IL‐18 [9, 47]. Persistent activation of the NLRP3 inflammasome exacerbates inflammation, further impairing neurogenesis, whereas its inhibitor, MCC950, reactivates neurogenesis in the hippocampus and SVZ [47]. Clearing microglia/macrophages from the ischemic zone reverses their inhibitory effects on neurogenesis [9]. Consequently, finely regulating their polarization state and promoting M2‐type conversion are key strategies for promoting neurogenesis after ischemic stroke [48].

It is worth noting that most of the current evidence regarding the interaction between microglia and NSCs originates from rodent stroke models; however, there are significant interspecies differences between the neuroimmune microenvironments of rodents and humans. In human ischemic brain tissue, the dynamic polarization trajectories of microglia and macrophages and their precise effects on endogenous neurogenesis in humans have not yet been fully elucidated, constituting a major knowledge gap in clinical translational research.

### 4.2. Metabolic Reprogramming: Metabolic Regulation of NSC Fate

The self‐renewal and differentiation of NSC exhibit stage‐specific metabolic characteristics: the proliferative phase relies on glycolysis for rapid energy acquisition and biosynthetic precursors, whereas the differentiation phase requires oxidative phosphorylation to meet the energy demands of mature neurons [11]. Stroke severely disrupts this metabolic equilibrium, inducing metabolic reprogramming in NSC and consequently impairing neurogenesis. Hypoxic postconditioning (HPC) is an effective neuroprotective strategy that achieves metabolic conversion by reducing fatty acid oxidation levels and increasing glycolytic flux in NSC, thereby promoting cell proliferation and migration. This beneficial effect is counteracted by either fatty acid oxidation activators or glycolysis inhibitors [11], confirming the key role of metabolic regulation in neurogenesis. The restoration of mitochondrial function is crucial for the survival and maturation of newly generated neurons. Transplantation of human NSC effectively restores mitochondrial electron transport chain function in ischemic regions and the hippocampus, providing energy support for neurogenesis while creating a favorable metabolic microenvironment for the functional integration of new neurons [49]. Therefore, targeting metabolic pathways in NSC to correct postischemic metabolic imbalance is a key strategy for enhancing endogenous neuroregenerative potential.

### 4.3. Meninges and Choroid Plexus: Novel Microenvironmental Regulatory Centers for Neurogenesis

The meninges and choroid plexus were originally considered responsible for providing mechanical protection and maintaining cerebrospinal fluid homeostasis. However, recent studies have revealed that both the meninges and choroid plexus also serve as mediators in neuroimmune interactions, lymphoid‐like system perfusion, and NSC niche signaling. This evidence demonstrates that the meninges and choroid plexus are novel microenvironmental regulators for neurogenesis poststroke [10]. The meninges provide a suitable microenvironment for the proliferation and homing of neural stem/progenitor cells. The choroid plexus directly regulates the proliferation, migration, and differentiation fate of NSC by secreting signaling molecules such as growth factors and cytokines [10]. Furthermore, both structures can enhance poststroke lymphoid‐like system perfusion by modulating neuroimmune interactions, thereby clearing inflammatory mediators and neurotoxic metabolic waste from ischemic regions and mitigating neuroinflammation. This process indirectly fosters conditions conducive to neurogenesis [10]. This discovery expands the understanding of the NSC niche microenvironment and provides a novel theoretical foundation for developing innovative therapeutic strategies for stroke (Figure 3).

**Figure 3:**
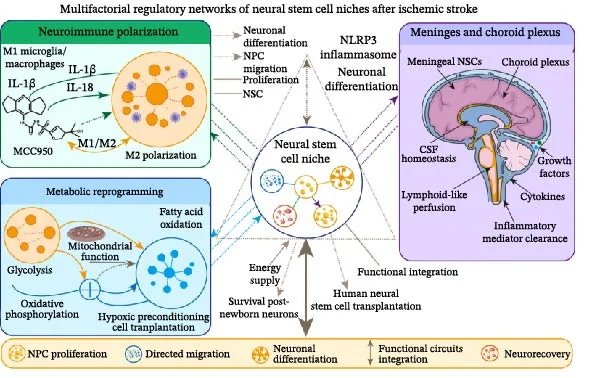
Multifactorial regulatory network of the neural stem cell (NSC) microenvironment following ischemic stroke. Following cerebral ischemia, three core microenvironmental modules act in concert to regulate endogenous neurogenesis. Within the neuroimmune module, M1‐type microglia/macrophages release IL‐1β/IL‐18 to activate the NLRP3 inflammasome, thereby inhibiting NSC proliferation, migration, and differentiation; the NLRP3 inhibitor MCC950 promotes M2 polarization, thereby restoring neurogenesis. The metabolic module regulates NSC fate via glycolysis, fatty acid oxidation, and mitochondrial oxidative phosphorylation, whilst hypoxia pretreatment or human NSC transplantation can correct metabolic dysfunction to support the survival of newborn neurons and the integration of neural circuits. The meningeal‐choroid plexus module secretes growth factors and cytokines, maintains cerebrospinal fluid homeostasis, and clears inflammatory mediators, thereby promoting neural regeneration. These axes work in concert to coordinate the proliferation, migration, and neuronal maturation of neural progenitor cells, thereby enabling the restoration of neural function.

## 5. Targeted Therapeutic Strategies for Promoting Neurogenesis Poststroke

Based on an in‐depth analysis of the mechanisms governing neurogenesis after ischemic stroke, three major categories of targeted therapeutic strategies have been developed: drug‐mediated regulation of endogenous NSC, exogenous stem cell transplantation, and nonpharmacological and biophysical interventions. Each strategy offers distinct advantages and has shown promise in neural repair in preclinical studies.

### 5.1. Pharmacological Targeting of Endogenous NSC

Targeted modulation of endogenous NSC using chemical drugs or natural compounds is a crucial therapeutic strategy for ischemic stroke. This approach offers the advantages of operational simplicity and high specificity. Natural compounds, particularly active components from traditional Chinese medicine, have received increased attention owing to their multitargeted effects and low toxicity. Traditional Chinese medicine monomers such as danphenolic acid A, phenylethanol glycosides, and hydroxycrocin A can promote NSC proliferation, migration, and neuronal differentiation by targeting core pathways such as Wnt/β‐catenin, consequently mitigating neurological deficits [39, 50, 51]. Traditional Chinese herbal formulas and herb pairs exert multipathway, multitarget regulatory effects, indirectly promoting endogenous neurogenesis while exerting anti‐inflammatory, anti‐apoptotic, and proangiogenic effects [52, 53]. Despite the considerable therapeutic potential exhibited by these natural compounds, their clinical translation remains limited owing to incomplete pharmacodynamic and pharmacokinetic studies and unoptimized administration routes and therapeutic windows. Rigorous long‐term safety assessments are required to ensure the efficacy and reliability of these compounds in the clinic.

### 5.2. Exogenous Stem Cell Transplantation

Exogenous stem cell transplantation exerts neuroprotective effects through the dual mechanisms of cell replacement and paracrine signaling, serving as a crucial strategy for directly replenishing neuronal sources in ischemic regions. Studies have primarily focused on NSC, mesenchymal stem cells, and induced pluripotent stem cells. Among these cell types, NSC are considered ideal seed cells for transplantation owing to their capacity for direct differentiation into neurons. After transplantation into the brains of animal models of stroke, human NSC can survive, migrate, and differentiate into functional neurons within the ischemic zone, thereby improving neurological function [54]. However, the hostile microenvironment within the ischemic zone reduces the survival rate of transplanted cells. Pretreatment strategies, such as nanoparticle‐based drug delivery systems or particles loaded with tanshinone IIA, can significantly enhance the survival, differentiation efficiency, and endogenous neuroprotective capacity of transplanted cells [55, 56]. Meanwhile, grafted NSCs modified by layer‐by‐layer (LbL) assembly (LbL‐NSCs) using gelatin and hyaluronic acid (HA) expedite the rehabilitative ability of engrafted NSCs following stroke [57]. In addition to chemical and nanoparticle‐based pretreatment, biophysical stimulation (such as electrical stimulation) prior to cell transplantation is another highly promising pretreatment method for donor NSCs. Pretransplant electrical stimulation can remodeling the transcriptomic profile of stem cells, enhancing cell survival, migratory capacity, and therapeutic efficacy following transplantation into damaged brain tissue [58]. This physical pretreatment strategy offers an alternative cell engineering approach to improving the efficacy of NSC transplantation. Nevertheless, prior to clinical application, the optimal stimulation parameters, cell‐type‐specific responses, and long‐term biosafety require further preclinical validation.

Notably, paracrine signaling is a core action mechanism of exogenous stem cell transplantation. Extracellular vesicles and exosomes secreted by transplanted cells can modulate the host brain microenvironment by alleviating neuroinflammation, protecting the neurovascular unit, and promoting angiogenesis and neurogenesis [59–61]. Furthermore, functional mitochondria derived from mesenchymal stem cells can be internalized by ischemic cells, promoting cell survival by reducing reactive oxygen species levels [61]. Mesenchymal stem cells have received increased attention owing to their accessibility and low immunogenicity. Future studies should focus on optimizing cell transplantation strategies, enhancing cell survival rates, and identifying key targets for paracrine signaling.

Although promising preclinical results have been observed in rodent and large animal models, there remain significant differences between animal studies and human clinical observations. Many of the reported benefits of NSC transplantation may stem from the remodeling of the paracrine microenvironment rather than direct neuronal replacement. Furthermore, there are considerable variations across different preclinical studies in terms of cell source, cell dose, timing of transplantation, and immunosuppression regimens, which hinder direct cross‐comparisons between studies.

### 5.3. Nonpharmacological and Biophysical Interventions for Neurogenesis

Owing to their noninvasive or minimally invasive nature and minimal side effects, nonpharmacological and biophysical interventions are increasingly evaluated for their efficacy in promoting neurogenesis after ischemic stroke. These interventions primarily include traditional Chinese therapies such as electroacupuncture and biophysical approaches such as hypoxic preconditioning and repetitive transcranial magnetic stimulation. These strategies mimic endogenous protective mechanisms to modulate the biological behavior of NSC, thereby facilitating neurogenesis [12]. Hypoxic preconditioning enhances the tolerance of NSC to ischemic stress and increases the survival rate of transplanted stem cells. Physical interventions such as electrical stimulation may also be used for stem cell preconditioning to enhance therapeutic efficacy [62]. The voltage‐gated potassium channel Kv4.2 activates multiple signaling pathways to upregulate BDNF expression, thereby promoting NSC proliferation and differentiation and improving sensory, motor, and cognitive functions after ischemic stroke [63]. However, the precise action mechanisms, optimal time windows, and protocol parameters of these strategies remain unclear. Further optimization of intervention protocols and clarification of action mechanisms may facilitate the development of safer, more effective neuroregenerative therapies. At present, across various preclinical studies, there remains a lack of uniform standards regarding the optimal stimulation parameters, therapeutic time window, and potential cellular mechanisms underlying electroacupuncture and repetitive transcranial magnetic stimulation; further standardized studies in large animal models are required before reliable clinical application can be undertaken (Figure 4).

**Figure 4:**
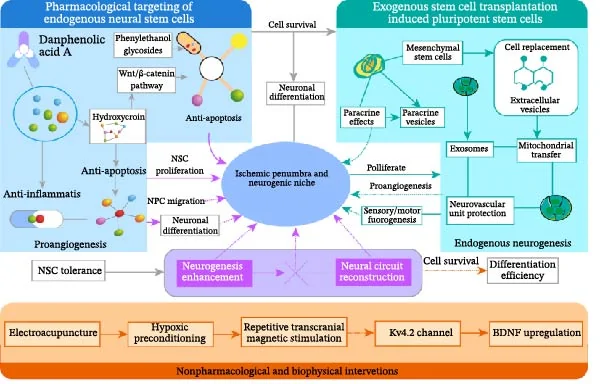
Three core therapeutic strategies for promoting endogenous neurogenesis following ischemic stroke. Chinese herbal monomers (danphenolic acid A, phenethyl glycoside, and hydroxycurcumin A) inhibit inflammation and apoptosis, promote angiogenesis, and stimulate the proliferation, migration, and neuronal differentiation of neural stem cells by modulating the Wnt/β‐catenin pathway. iPSC and mesenchymal stem cell transplantation exert their effects through cellular replacement and paracrine mechanisms. Released exosomes and extracellular vesicles protect neurovascular units and promote angiogenesis; mitochondrial transfer enhances the survival and differentiation capacity of transplanted cells. Nonpharmacological interventions (electroacupuncture, hypoxic preconditioning, and rTMS) upregulate brain‐derived neurotrophic factor (BDNF) by activating the Kv4.2 channel and remodeling the neurogenic microenvironment. All therapies target the ischemic penumbra to enhance neurogenesis and reconstruct neural circuits.

## 6. Challenges in Neurogenesis Poststroke and Prospects for Clinical Translation

Despite extensive research on the regulatory mechanisms of neurogenesis and the development of various therapeutic strategies poststroke, numerous challenges persist in translating basic research into clinical practice. Multimodal combination therapies and novel technological applications are key directions for future research and clinical translation.

### 6.1. Controversy and Validation Regarding the Correlation Between Neurogenesis and Functional Recovery

Animal studies have confirmed that promoting neurogenesis improves poststroke behavioral outcomes, suggesting that neurogenesis forms a crucial foundation for functional recovery. However, the direct causal relationship between newly generated neurons and functional restoration remains unresolved. Key points of contention persist regarding the long‐term survival rate of these neurons, the efficiency of their functional integration, and their specific contribution to the reconstruction of neural circuits [63, 64]. Deletion of the long noncoding RNA H19 inhibits neurogenesis and impairs spontaneous functional recovery in mice, indicating that the fate and functional integration of newly generated neurons are crucial determinants of repair efficacy [65]. Direct observation and quantification of neurogenesis in patients with stroke are exceedingly challenging. Previous studies have relied on indirect imaging markers or postmortem tissue analysis, both of which hinder dynamic monitoring and quantitative assessment [10] and consequently limit the clinical translation of preclinical findings. Therefore, developing noninvasive, dynamic monitoring techniques for human neurogenesis is necessary. Concurrently, precision tools such as optogenetics and chemogenetics should be used in animal studies to elucidate the specific functions of new neurons in neural circuit reconstruction in establishing a direct causal relationship. Understanding this relationship is a crucial prerequisite for the clinical translation of fundamental research.

Collectively, multiple unresolved knowledge gaps remain in this field. First, most mechanistic conclusions are based on preclinical rodent data, and translating these findings into human pathophysiology faces substantial barriers. Second, conflicting results are frequently reported across different laboratories regarding the magnitude of poststroke human neurogenesis, which can be partially explained by differences in animal stroke models, injury severity, animal age, sampling time‑points, and detection methodologies. Third, the causal contribution of endogenously‑born new neurons to long‑term functional recovery in human stroke patients is still unproven. Therefore, caution should be exercised when extrapolating encouraging preclinical observations directly to clinical expectations. Most mechanistic insights discussed throughout this review originate from rodent models, and interspecies differences must be carefully considered when translating preclinical observations toward human stroke therapy.

### 6.2. Treatment Window Management and Personalized Strategies

The brain microenvironment markedly varies across distinct pathological phases after stroke, exerting different regulatory effects on neurogenesis. Precise control of the therapeutic time window is important for clinical translation. The acute phase of ischemic stroke is characterized by intense cerebral inflammation and oxidative stress, wherein excessive activation of neurogenesis may exacerbate the injury. However, the recovery phase is characterized by a stabilizing microenvironment that is more conducive to NSC migration, differentiation, and functional integration of new neurons. The therapeutic effects of Rac1 exhibit pronounced time‐dependent characteristics [29]. Furthermore, different therapeutic approaches present unique safety challenges. For instance, exogenous stem cell transplantation carries the risks of immune rejection, tumorigenicity, low cell survival rates, and suboptimal directed differentiation efficiency [66], whereas pharmacological interventions necessitate vigilance for off‐target effects, long‐term safety concerns, and potential impacts on normal intracerebral neurogenesis. In addition, patient‐specific factors, such as age, stroke type, and comorbidities, significantly influence neurogenesis efficiency and therapeutic outcomes. It is worth noting that aging, as a major confounding factor, profoundly reshapes the neurogenic response following a stroke. Ischemic stroke primarily affects the elderly population, and aging itself impairs the maintenance of the resting NSC pool, reduces the proliferative and migratory capacity of NSCs, disrupts the balance between neuronal and glial differentiation, and reshapes the brain’s inflammatory microenvironment into a pro‐inflammatory, antiregenerative state. Age‐related preactivation of microglia further exacerbates postischemic neuroinflammation, thereby inhibiting endogenous neurogenesis and diminishing the therapeutic benefits of regenerative interventions. Most preclinical mechanistic data originate from young experimental animals; these data fail to fully replicate the deteriorated neurogenic microenvironment observed in elderly human patients. Therefore, in future preclinical studies, priority should be given to age‐stratified experimental designs and age‐matched animal models to better facilitate the transition from the laboratory to the clinic [67, 68]. Consequently, future approaches must integrate disease stages with individual patient characteristics to achieve therapeutic strategies that balance the activation of endogenous repair mechanisms with the prevention of adverse effects.

### 6.3. Prospects for Multimodal Combination Therapy and Novel Technologies

The use of a single therapeutic strategy is insufficient for addressing multiple challenges following stroke, including neural damage, inflammatory responses, and vascular disruption. The close coupling between neurogenesis and angiogenesis provides a theoretical foundation for developing multimodal combination therapies. Future studies should focus on integrating neurogenesis‐promoting strategies with vascular recanalization, rehabilitation training, and neuromodulation techniques to facilitate structural and functional brain repair through synergistic effects. Repetitive transcranial magnetic stimulation can directly promote neurogenesis while indirectly ameliorating the inflammatory microenvironment [44, 69], and electroacupuncture combined with polygala saponins can synergistically enhance the reparative efficacy of stem cell transplantation [70]. Emerging technologies hold promise for overcoming existing therapeutic limitations, offering novel approaches for neurogenesis regulation and neural repair. For instance, cell‐free therapies based on extracellular vesicles/exosomes mitigate the risks of tumorigenicity and immune rejection associated with stem cell transplantation [4, 60, 71]. Gene editing technologies can precisely regulate endogenous NSC signaling pathways to enhance neurogenesis efficiency. Three‐dimensional (3D) bioprinting enables the construction of biomimetic neural tissue scaffolds that provide optimal microenvironments for NSC. In addition, biomaterial‐mediated drug delivery systems can facilitate the sustained release of growth factors to activate and recruit endogenous neural stem/progenitor cells [72]. The deep integration of these emerging technologies with conventional therapeutic strategies may provide safer, more efficient, and precise treatments for stroke.

## 7. Conclusion and Future Perspectives

This review provides a systematic overview of the entire process of endogenous neurogenesis triggered by ischemic stroke, including the activation and proliferation of NSCs, the directed migration of neural progenitor cells, and, ultimately, neuronal differentiation and synaptic integration. We provide a comprehensive analysis of this multilevel regulatory network, covering core signaling cascades (Wnt/β‐catenin, PI3K/Akt, and ASIC1a/RhoA), factors in the ischemic penumbra microenvironment, including acidosis and microglial polarization, and microenvironmental modulators such as the meninges and choroid plexus. This article summarizes three main therapeutic approaches, including small‐molecule pharmacological modulation, stem cell therapy, and noninvasive neuromodulation. Two clinically repurposed drugs—ambroxol and ASIC1a antagonists—have emerged as highly promising candidates for translational medicine: ambroxol promotes neuronal maturation via the GCase/Wnt/β‐catenin axis, whilst ASIC1a antagonists alleviate acidosis‐induced migration impairments in NPCs, providing new intervention targets for poststroke neural repair.

Although a wealth of preclinical evidence has demonstrated the reparative potential of endogenous neurogenesis, numerous obstacles remain in translating basic research findings into clinical practice. Notably, we consider that the current field still faces prominent controversies and unresolved contradictions that severely hinder clinical transformation. First, the precise causal relationship between newly generated neurons and long‐term neurological recovery has not yet been fully validated; this is primarily attributable to the lack of noninvasive biomarkers of neurogenesis in humans, as well as the limited ability of rodent models to replicate the pathophysiology of human cerebrovascular disease. A central point of contention in this field is whether newly generated neurons can actually integrate functionally into damaged human neural circuits, or whether they merely represent a compensatory, nonfunctional proliferative response following a stroke; to date, no consensus has been reached on this matter. Second, the ischemic brain microenvironment undergoes a significant temporal shift from an acute inflammatory state to one that promotes recovery; premature activation of neurogenesis during the acute phase may exacerbate secondary damage, whilst individual variations—such as patient age and stroke severity—further necessitate the development of personalized intervention strategies. The absence of a standardized time‐window intervention model has led to inconsistent treatment outcomes across different preclinical studies, which also constitutes a major point of contention in this field. Furthermore, monotherapy cannot simultaneously address issues such as neuronal loss, inflammatory infiltration, and metabolic dysfunction, whilst stem cell transplantation still faces practical risks such as low graft survival rates and potential oncogenicity, which limit its large‐scale clinical promotion.

From our critical perspective, we argue that the greatest bottleneck for translating NSC‐mediated neurogenesis into stroke treatment is not the lack of regulatory targets, but the disconnection between preclinical animal research and individualized human pathological characteristics, as well as the absence of precise, staged, and safe intervention systems. In response to the aforementioned challenges in translating research into clinical practice and to advance the clinical application of endogenous neurogenesis therapies, future research must draw upon the temporal patterns of poststroke neurogenesis and the brain microenvironment to establish a precise, phased, and multimodal integrated intervention system.

First, a time‐sequenced, targeted treatment regimen should be established. For example, during the acute phase of stroke, ASIC1a antagonists may be used to alleviate cerebral acidosis, lift the inhibitory effects of the microenvironment on neural progenitor cell migration, mitigate secondary brain damage, and preserve endogenous repair potential. Meanwhile, during the subacute repair window, Wnt pathway agonists, such as ambroxol, may be administered to stably activate neurogenesis, promote neuronal differentiation and maturation, and expand the pool of functional newborn neurons. Throughout the entire process, noninvasive neuromodulation techniques should be integrated to enhance synaptic plasticity and neural circuit integration, thereby achieving synergistic effects across the full cycle of neural repair. Second, by leveraging cutting‐edge biotechnologies to overcome clinical translation bottlenecks, liquid biopsy biomarkers and specific molecular imaging probes need to be developed to enable noninvasive, real‐time monitoring of neurogenesis in humans and quantitative assessment of therapeutic efficacy. And, combined with technologies such as CRISPR gene editing and bionic neural scaffolds should be performed to reshape the microenvironment of the ischemic penumbra, stabilize the homeostasis of NSCs, and enhance the survival and integration efficiency of newly generated neurons. Finally, a standard preclinical evaluation system needs to be established. Utilizing large‐animal models that closely mimic the pathological characteristics of human cerebrovascular disease, the safety and efficacy of combined intervention protocols will be systematically validated, bridging the translational gap between basic research and clinical application, and facilitating the clinical implementation of neuro‐repair therapies for stroke. We believe that the current over‐reliance on rodent models is the root cause of the poor reproducibility of neurogenesis therapies; future research should prioritize the establishment of a standardized evaluation system for large animals in order to resolve the existing academic controversies.

In summary, endogenous neurogenesis is the core endogenous driving force behind neurological recovery following a stroke. From our balanced and critical perspective, whilst neurogenesis mediated by NSCs holds immense reparative potential in the treatment of ischemic stroke, its current limitations—including unclear clinical causality, controversy surrounding the therapeutic window, and insufficient translational safety—cannot be overlooked. Precision‐based, multimodal, and personalized intervention strategies designed to target the entire neurogenesis process can effectively stimulate the self‐repair potential of the adult brain. With continuous advances in molecularly targeted therapies and smart neuromodulation technologies, neurogenesis‐based stroke rehabilitation strategies will gradually transition from preclinical exploration to standard clinical practice, providing novel and effective solutions to improve the long‐term prognosis of stroke patients and alleviate the global burden of stroke‐related disability.

## Author Contributions

Anyong Yu and Hongfei Ge proposed this idea. Peng Wang, Peng Ye, and Jin Wu were responsible for reviewing the relevant literature. Lili Shi and Xuheng Jiang were responsible for drawing the mechanism diagrams. Shuhong Wang and Ji Zhang wrote the first draft of the article. Anyong Yu, Hongfei Ge, and Yuanlan Lu reviewed and finalized the manuscript.

## Funding

This research was supported by grants obtained from the National Natural Science Foundation of China (Grants 82260254 and 82271424), the Guizhou Provincial Department of Science and Technology Science and Technology Program Project (Grant Qian Ke He Ji Chu‐ZK [2023]), and the Guizhou Province Clinical Research Center for Emergency Medicine (Grant LCZX [2025]004).

## Disclosure

All authors participated in the discussions and approved the final version of the article.

## Ethics Statement

The authors have nothing to report.

## Consent

The authors have nothing to report.

## Conflicts of Interest

The authors declare no conflicts of interest.

## Data Availability

No new data were created or analyzed in this study.
